# Knockdown of *miR-21 *in human breast cancer cell lines inhibits proliferation, *in vitro *migration and *in vivo *tumor growth

**DOI:** 10.1186/bcr2803

**Published:** 2011-01-10

**Authors:** Li Xu Yan, Qi Nian Wu, Yan Zhang, Yang Yang Li, Ding Zhun Liao, Jing Hui Hou, Jia Fu, Mu Sheng Zeng, Jing Ping Yun, Qiu Liang Wu, Yi Xin Zeng, Jian Yong Shao

**Affiliations:** 1 State Key Laboratory of Oncology in Southern China, Sun Yat-Sen University Cancer Center, 651 Dong Feng Road East, Guangzhou, 510060, PR China; 2 Department of Pathology, Sun Yat-Sen University Cancer Center, 651 Dong Feng Road East, Guangzhou, 510060, PR China; 3 Department of Experiment Research, Sun Yat-Sen University Cancer Center, 651 Dong Feng Road East, Guangzhou, 510060, PR China

## Abstract

**Introduction:**

MicroRNAs (miRNAs) are a class of small non-coding RNAs (20 to 24 nucleotides) that post-transcriptionally modulate gene expression. A key oncomir in carcinogenesis is *miR-21*, which is consistently up-regulated in a wide range of cancers. However, few functional studies are available for *miR-21*, and few targets have been identified. In this study, we explored the role of *miR-21 *in human breast cancer cells and tissues, and searched for *miR-21 *targets.

**Methods:**

We used *in vitro *and *in vivo *assays to explore the role of *miR-21 *in the malignant progression of human breast cancer, using *miR-21 *knockdown. Using LNA silencing combined to microarray technology and target prediction, we screened for potential targets of *miR-21 *and validated direct targets by using luciferase reporter assay and Western blot. Two candidate target genes (EIF4A2 and ANKRD46) were selected for analysis of correlation with clinicopathological characteristics and prognosis using immunohistochemistry on cancer tissue microrrays.

**Results:**

Anti-miR-21 inhibited growth and migration of MCF-7 and MDA-MB-231 cells *in vitro*, and tumor growth in nude mice. Knockdown of *miR-21 *significantly increased the expression of *ANKRD46 *at both mRNA and protein levels. Luciferase assays using a reporter carrying a putative target site in the 3' untranslated region of *ANKRD46 *revealed that *miR-21 *directly targeted *ANKRD46*. *miR-21 *and EIF4A2 protein were inversely expressed in breast cancers (r_s _= -0.283, *P *= 0.005, Spearman's correlation analysis).

**Conclusions:**

Knockdown of *miR-21 *in MCF-7 and MDA-MB-231 cells inhibits *in vitro *and *in vivo *growth as well as *in vitro *migration. *ANKRD46 *is newly identified as a direct target of *miR-21 *in BC. These results suggest that inhibitory strategies against *miR-21 *using peptide nucleic acids (PNAs)-antimiR-21 may provide potential therapeutic applications in breast cancer treatment.

## Introduction

Breast cancer (BC) is by far the most frequent cancer of women (23% of all cancers), with an estimated 1.15 million new cases worldwide in 2002 [1]. It is still the leading cause of cancer mortality in women [1]. Despite research and resources dedicated to elucidating the molecular mechanisms of BC, the precise mechanisms of its initiation and progression remain unclear.

MicroRNAs (miRNAs) are small non-coding RNAs (20 to 24 nucleotides) that post-transcriptionally modulate gene expression by negatively regulating the stability or translational efficiency of their target mRNAs [2]. After the discovery of miRNAs, and findings indicating that they play a role in cancer, the concept of "oncomirs" was proposed [3]. In particular, *miR-21 *[miRBase: MIMAT0000076] has emerged as a key oncomir, since it is the most consistently up-regulated miRNA in a wide range of cancers [4-7].

Functional studies showed that knockdown of *miR-21 *in MCF7 cells led to reduced proliferation and tumor growth [8,9]. Knockdown of *miR-21 *in MDA-MB-231 cells significantly reduced invasion and lung metastasis [10]. These data clearly implicate *miR-21 *as a key molecule in carcinogenesis, but functional studies that demonstrate cause and effect relationships between *miR-21 *and target genes are lacking. Given that miRNAs usually target multiple genes post-transcriptionally, *miR-21 *is likely to exert its effects by regulating many genes involved in BC.

The inhibition of miRNAs using antisense oligonucleotides (ASOs) is a unique and effective technique for investigating miRNA functions and targets. Peptide nucleic acids (PNAs) are artificial oligonucleotides constructed on a peptide-like backbone. PNAs have a stronger affinity and greater specificity for DNA or RNA than natural nucleic acids, and are resistant to nucleases [11]. PNA-based ASOs can be used without transfection reagents, and are highly effective and sequence-specific. They provide long-lasting inhibition of miRNAs, and show no cytotoxicity up to 1 μM [11]. Therefore, we used a PNA *miR-21 *inhibitor for *in vivo *investigation.

In this study, we explored the role of *miR-21 *in the malignant progression of human BC by assaying *in vitro *and *in vivo *function after *miR-21 *knockdown. We also searched for *miR-21 *targets using gene prediction-based and systematic screening approaches. Two potential target genes eukaryotic translation initiation factor 4A2 (*EIF4A2*) [NCBI: NM001967] and ankyrin repeat domain 46 (*ANKRD46*) [NCBI: NM198401] were selected for correlation analysis between protein levels and clinicopathological characteristics as well as prognosis using immunohistochemistry (IHC) on cancer tissue microrrays (TMAs).

## Materials and methods

### Tissue specimens and TMAs construction

*In situ *hybridization analysis was performed on fresh samples from BC or fibroadenoma (FA) tissues with paired normal adjacent tissues (NATs, > 2 cm from tumor tissues) obtained from Sun Yat-sen University Cancer Center (SYSUCC) (Guangzhou, China) between January and March 2009. For IHC staining of *miR-21 *predicted target genes, formalin-fixed paraffin-embedded tissues were obtained from 99 randomly selected BC patients without neoadjuvant therapy at SYSUCC from January 2000 to November 2004, from whom informed consent and agreement, and clinicopathological information was available. A pathologist reviewed slides from all blocks, selecting representative areas of invasive tumor tissue to be cored. Selected cores were analyzed in duplicate using a MiniCore Tissue Arrayer (Alphelys, Passage Paul Langevin, Plaisir, France) with a 1-mm needle. The diagnosis and histological grade of each case were independently confirmed by two pathologists based on World Health Organization classification [12]. The clinical stage was classified according to the American Joint Committee on Cancer (AJCC) tumor-lymph node-metastasis (TNM) classification system [13]. The study was approved by the Research Ethics Committee of SYSUCC (Reference number: YP-2009168). The clinicopathological characteristics and follow-up data of the patients are summarized in Table 1.

**Table 1:** Clinicopathological characteristics and follow-up data for 99 patients with BC

Characteristics	Number of patients/Number analyzed (%)
Median age (range)	48 (30 to 74) (years)
Histological type*	
Ductal	93/99 (94%)
Lobular	1/99 (1%)
Other	5/99 (5%)
Histological grade*	
I	22/99 (22%)
II	58/99 (58%)
III	19/99 (20%)
Lymph node status at time of primary diagnosis	
Metastasis	57/99 (58%)
No metastasis	42/99 (42%)
AJCC clinical stage**	
I	8/99 (8%)
II	68/99 (69%)
III	23/99 (23%)
Overall survival (median, range)	74 (6 to 112) (months)
Alive without evidence of cancer	45/99 (68%)
Alive with cancer	14/99 (14%)
Died of cancer	40/99 (40%)
Died of other disease	0/99 (0%)

* According to the WHO classification of BC. ** According to the AJCC staging system; AJCC, American Joint Committee on Cancer; BC, breast cancer; WHO, World Health Organization.

### Locked nucleic acid (LNA)-based *in situ *hybridization for miRNA

To study the spatial and temporal expression of miRNAs with high sensitivity and resolution, the miRNA chromogenic *in situ *hybridization (CISH) and fluorescein *in situ *hybridization (FISH) protocol [14] were optimized (Additional file 1).

### Transfection of LNA-antimiR-21 into BC cells

MCF-7 and MDA-MB-231 cells were maintained in Dulbecco's modified Eagle's medium, supplemented with 100 U/ml penicillin, 100 μg/ml streptomycin and 10% fetal bovine serum (GIBCO-Invitrogen, Carlsbad, CA, USA). For transfection, the LNA-antimiR-21 or LNA-control (Exiqon A/S, Skelstedet, Vedbaek, Denmark) were delivered at a final concentration of 50 nM using Lipofectamine 2000 reagent (Invitrogen).

### 3-(4,5-dimethylthiazol-2-yl)-2,5-diphenyltetrazolium bromide (MTT) assay and colony formation assay

Growing cells (2 × 10^3 ^cells per well) were seeded into 96-well plates. At 24 h after LNA-transfection, cells were stained with 20 μl sterile MTT dye (5 mg/ml; Sigma-Aldrich Corp, St. Louis, MO, USA), followed by 4 h at 37°C. After supernatant removal, 150 μl of dimethyl sulphoxide (Sigma) was added and thoroughly mixed for 15 minutes. Absorbence was measured with a microplate reader (SpectraMax M5; Molecular Devices Corp., Silicon Valley, CA, USA) at 490 nm. For colony formation assays, cells were seeded in six-well plates (0.5 × 10^3 ^cells per well) and cultured for two weeks. Colonies were fixed with methanol for 10 minutes and stained with 1% crystal violet (Sigma) for 1 minute. Each cell group was measured in triplicate.

### Wound healing assay

Cells cultured in the presence of 50 nM LNA-antimiR-21 or LNA-control for 24 h were allowed to reach confluence before dragging a 1-mL sterile pipette tip (Axygen Scientific, Inc., Union City, CA, USA) through the monolayer. Cells were washed to remove cellular debris and allowed to migrate for 24 h or 48 h. Images were taken at time 0 h, 24 h and 48 h post-wounding using a digital camera system (Leica DFC 480; Leica Microsystems, Bannockburn, IL, USA). The motility of the cells was determined as repaired area percentage [15]. Each cell group was measured in triplicate.

### Validation of tumor growth-promoting activity of *miR-21 *in an animal model

Five- to six-week-old female BALB/c-nude mice (Slaccas Shanghai Laboratory Animal Co., Ltd., Shanghai, China) were used for experimental tumorigenicity assays. To facilitate estrogen-dependent xenograft establishment, each mouse received 17-estradiol (20 mg/kg; Sigma) intraperitoneally once a week. One week after treatment, equivalent amounts of MCF-7 cells, treated with PNA-antimiR-21 or PNA-control (100 nM for 48 h; Panagene, Inc., Yuseong-gu, Daejeon, Korea) without transfection reagents according to the manufacturer's protocol, were injected subcutaneously (10^7 ^cells/tumor) into the left axilla of nude mice [16]. Mice were weighed, and tumor width (W) and length (L) were measured every day. Tumor volume was estimated according to the standard formula: V = ∏/6 × L × W^2^, as described previously [17]. Animals were killed nine days after initial growth of the MCF-7 xenografts was detectable, and tumors were extracted. In all experiments, the ethics guidelines for investigations in conscious animals were followed, with approval from the local Ethics Committee for Animal Research.

### mRNA array and data mining

MCF-7 and MDA-MB-231 cells were transfected either with LNA-antimiR-21 or with LNA-control at a final concentration of 50 nM. Total RNAs were isolated from MCF-7 cells 48 h post transfection and from MDA-MB-231 cells 36 h post transfection, respectively, using Trizol Reagent (Invitrogen). The mRNA expression profile was performed using human genome oligo array service V1.0 (Catalog Number 400010; CapitalBio, Beijing, China) as described [18]. Each sample was analyzed once, and the CapitalBio data preprocess, normalization and filtering were as previously described [18]. Ratios were defined as marginal signal intensity when there was a substantial amount of variation in the signal intensity within the pixels from 800 to 1,500. All the microarray data have been deposited to the Gene Expression Omnibus (GEO) [19] and are accessible through GEO Series accession number [GEO: GSE20627].

### Relative quantitative reverse transcription-polymerase chain reaction (qRT-PCR)

For validation of mRNA array and quantitative analysis of *miR-21 *as well as potential target genes, qRT-PCR was used as previously described [20]. The primers for qRT-PCR are in Additional file 2. The relative expression was calculated using the equation relative quantification (RQ) = 2^-^ΔΔ^CT ^[21].

### Computational prediction of *miR-21 *target genes

Predicted *miR-21 *targets were identified using the algorithms of TargetScan 5.1 [22], miRBase Targets V5 [23], miRNAMap 2.0 [24], PicTar [25] and miRanda 3.0 [26].

### Luciferase reporter assay

The 3' untranslated region (3' UTR) of mRNA sequence of *ANKRD46 *containing predicted *miR-21 *binding site was amplified by PCR. PCR primers were listed in Additional file 2. After amplification, PCR products were cloned into the pMIR-REPORT (Applied Biosystems, Foster City, CA, USA), resulting in the pMIR-REPORT-3'*ANKRD46*. Mutation of *ANKRD46 *was introduced in the predicted *miR-21 *binding site by a QuikChange site-directed mutagenesis kit (Stratagene, Foster City, CA, USA). Wild-type *EIF4A2 *and mutant *EIF4A2 *were cloned into pMD19-T Simple Vector by TaKaRa Biotechnology CO., LTD. (Dalian, Liaoning, China) and then were individually subcloned downstream of the luciferase coding sequence in the pMIR-REPORT (Applied Biosystems). All constructs were verified by DNA sequencing.

For reporter assays, wide-type or mutant reporter constructs (15 ng) were cotransfected into 293T cells in twelve-well plates with *miR-21 *or miR-control (50 nM; GenePharma, Shanghai, China) and Renilla plasmid (5 ng) using lipofectamine 2000 (Invitrogen). Firefly and Renilla luciferase activities were measured by using a Dual Luciferase Assay (Promega, Madison, WI, USA) 24 h after transfection. Firefly luciferase values were normalized to Renilla, and the ratio of firefly/renilla was presented.

### Immunoblot analysis

Cells were harvested and lysed in radioimmune precipitation buffer (Upstate, Lake Placid, NY, USA) at the indicted time post-transfection. Antibodies used for immunoblot analysis were against ANKRD46 (1:500 dilution; sc-87548, Santa Cruz Biotechnology, Inc., Santa Cruz, CA, USA), EIF4A2 (1:1000 dilution; ab31218, Abcam, Cambridge, UK) and GAPDH (1:3,000 dilution; sc-32233, Santa Cruz Biotechnology), as a loading control. All bands were detected using a SuperSignal West Pico Chemiluminescent Substrate (Pierce, Rockford, IL, USA).

### Immunohistochemical staining

IHC and scoring of the estrogen receptor (ER), progesterone receptor (PR) and CerbB2 were performed as previously described [20]. Slides were incubated with primary antibodies against ANKRD46 (1:150 dilution; sc-87548, Santa Cruz Biotechnology); or EIF4A2 (1:700 dilution; ab31218, Abcam). All slides were processed simultaneously in identical conditions per the manufacturer's instructions. Three observers independently determined consensus scoring of EIF4A2 and ANKRD46 immunostaining using a semi-quantitative estimation according to the percentage of positive cells and the intensity of staining as described previously [27]. With these data, the composite score was obtained by adding the values of the staining intensity and relative abundance [28]. Samples with scores lower than the median score were grouped as low protein expression [29].

### Statistical analysis

Spearman's rank correlation test was used for correlation analysis between predicted target gene protein levels and endogenous *miR-21 *levels measured previously by qRT-PCR [20]. Pearson's Chi-Square tests were used to compare target gene expression levels to clinicopathological characteristics. Survival curves were estimated by the Kaplan-Meier method and log-rank test. All analysis used SPSS 16.0 for Windows (SPSS Inc, Chicago, IL, USA). All tests were two-tailed, and the significance level was set at *P *< 0.05.

## Results

### *miR-21 *is overexpressed in BC tissues and cell lines

**Figure 1:**
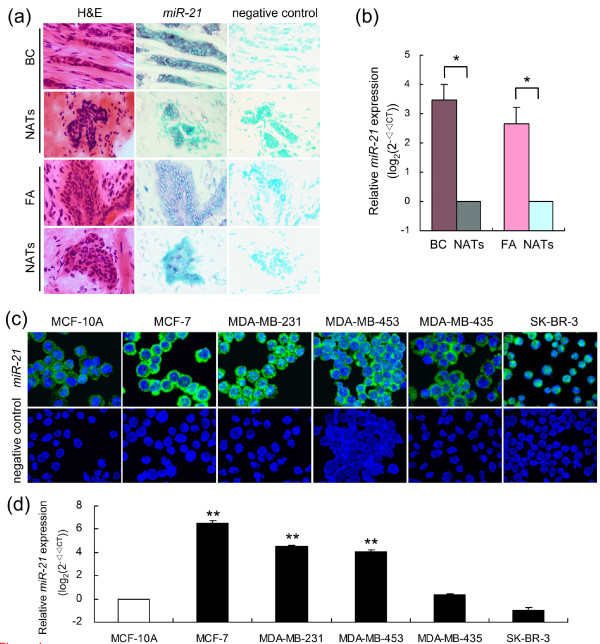
**Altered expression of *miR-21 *in different breast tumor types and breast cell lines**. **(a) **CISH detection using *miR-21 *LNA detection probe (blue) or scramble-miR as negative control were performed on consecutive 8 μM cryo-sections obtained from BC/FA tissues and corresponding NATs. DNA was counterstained with methyl green (green, × 400 magnification). **(b) **Total RNA was used to quantify *miR-21 *expression by relative qRT-PCR, normalizing on *U6 RNA *levels. The graph shows a log_2_-scale RQ calculated by normalizing the *miR-21 *expression values in the tumors on those in the NATs. Data indicate the mean (+SD) of four independent samples. * *P *< 0.05. **(c) **FISH detection of *miR-21 *in five BC cell lines (MCF-7, MDA-MB-231, MDA-MB-453, MDA-MB-435, SK-BR-3) and one non-tumorigenic epithelial cell line (MCF-10A). Positive *in situ *hybridization signals are visualized in green, while blue depicts DAPI nuclear stain (× 1,000 magnification). **(d) **qRT-PCR analysis show that MCF-7, MDA-MB-231 and MDA-MB-453 cells express higher levels of *miR-21 *compared with MCF-10A cells. Data are mean (+SD) of three replicates. ** *P *< 0.01; BC, breast cancer; FA, fibroadenoma; NATs, normal adjacent tissues.

Expression of *miR-21 *was detected in the cytoplasm in cancerous and luminal epithelial cells, and occasionally in fibroblasts. In BC patients, an increase in *miR-21 *staining intensity was observed in BC and FA tissues compared with corresponding NATs (Figure 1a). Parallel detection by FISH is shown in Additional file 3. Consistent with the CISH results, quantitative analysis indicated that *miR-21 *expression was significantly increased by 4.44- to 2.02-fold in BC tissues compared with NATs (*P *= 0.019, *n *= 4), and increased in FA tissues by 3.03- to 1.89-fold (*P *= 0.008, *n *= 4, Figure 1b). FISH and qRT-PCR were used to measure *miR-21 *levels in five BC cell lines (MCF-7, MDA-MB-231, MDA-MB-453, MDA-MB-435, and SK-BR-3) and one non-tumorigenic epithelial cell line (MCF-10A). Consistent with previous findings [8,10,30], *miR-21 *overexpressed in MCF-7, MDA-MB-231 and MDA-MB-453 cell lines from 6.48- to 4.04-fold compared with MCF-10A cell line (*P *< 0.01, Figure 1d).

### LNA-antimiR-21 inhibits BC cell growth, proliferation and migration *in vitro*

**Figure 2:**
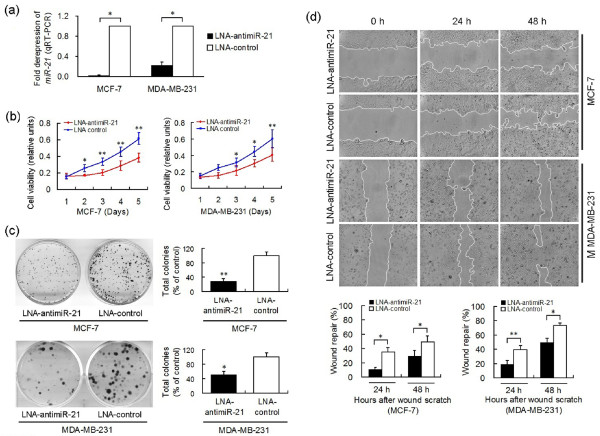
**LNA-antimiR-21 suppressed BC cell growth, proliferation and migration *in vitro***. **(a) ***miR-21 *expression levels (normalized to *U6 RNA*) were significantly depressed by 98% (*P *< 0.01) in MCF-7/LNA-antimiR-21 and 77% (*P *< 0.01) in MDA/LNA-antimiR-21 cells, relative to the LNA-control. The graph shows RQ calculated by normalizing the *miR-21 *expression values in LNA-antimiR-21 treated cells on those in the LNA-control treated cells. **(b) **MTT assay showed that MCF-7/antimiR-21 and MDA-MB-231/antimiR-21 cells grew slower than cells transfected with the LNA-control. **(c) **Representative wells showing total numbers of colonies formed by LNA-antimiR-treated cells standardized against control cells (set to 100%). **(d) **Representative image of *in vitro *wound healing assay of MCF-7 and MDA-MB-231 at 0, 24 and 48 h after wound scratch. Data are mean + SD, *n *= 3. * *P *< 0.05, ** *P *< 0.01.

MCF-7 and MDA-MB-231 cell lines were selected to investigate *miR-21 *functions and targets by using sequence-specific functional inhibition of *miR-21*, because both cell lines express higher levels of *miR-21 *compared with MCF-10A cells. Optimal doses and time points for transfection of LNA reagents were determined by evaluating *miR-21 *levels using qRT-PCR (Additional file 4). Knockdown of *miR-21 *reduced *miR-21 *levels by 98% in MCF-7 cells, and 77% in MDA-MB-231 cells (*P *< 0.01) (Figure 2a). LNA-antimiR-21 led to a decrease in MCF-7 cell growth (Figure 2b) and proliferation (29%, *P *= 0.003, Figure 2c). Similar inhibition of cell growth and proliferation effects (51%, *P *= 0.011, Figure 2c) was also observed in MDA/LNA-antimiR-21 cells (data not shown). *In vitro *wound healing assays showed that wound repair in MCF/LNA-antimiR-21 and MDA/LNA-antimiR-21 was delayed compared with MCF/LNA-control and MDA/LNA-control cells (data not shown). Knockdown of *miR-21 *suppressed MCF-7 cell migration by up to 69% (*P *= 0.013), and MDA-MB-231 migration by 51% (*P *= 0.001), compared with the LNA-control at 24 h after wound scratch (Figure 2d). These data demonstrate the tumorigenic properties of *miR-21 *in regulating cell growth, proliferation and migration.

### PNA-antimiR-21 inhibits tumor growth *in vivo*

**Figure 3:**
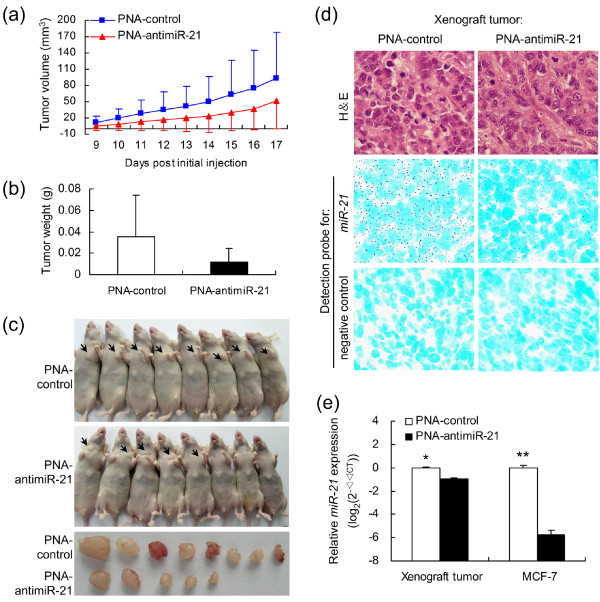
**Effect of miR-21-knockdown on MCF-7 cells growth in nude mice: *in vivo *functional studies**. MCF-7 cells (10^7 ^cells/tumor), treated with PNA-antimiR-21 or PNA-control (100 nM for 48 h) without transfection reagents, were injected subcutaneously into the left axilla of nude mice. **(a) **Growth curves for MCF/PNA-antimiR-21 (*n *= 5) vs. MCF/PNA-control (*n *= 8) cells in an *in vivo *proliferation assay. All *P-*values > 0.05. **(b) **Tumors were weighed after animals were killed at 17 days post-tumor-cell injection. Decreasing trend for both number and size of tumors from MCF/PNA-antimiR-21 compared with MCF/PNA-control group, although differences were not significant (*P *= 0.065, Mann-Whitney test). **(c) **Mice and tumors extracted from MCF/PNA-antimiR-21 and MCF/PNA-control groups. **(d) **Representative photomicrographs of CISH for *miR-21 *on xenograft tumor sections obtained from mice bearing MCF/PNA-antimiR-21 or MCF/PNA-control groups (× 400). **(e) **qRT-PCR analysis of *miR-21 *expression in MCF-7 cells 48 h post PNA-treatment and in xenograft tumors after mice sacrifice. Data in (a), (b) and (e) indicate the mean + SD. * *P *< 0.05, ** *P *< 0.01. PNA, peptide nucleic acid.

To address the potential effects of PNA-antimiR-21 *in vivo *on the growth of BC cells, equal numbers (3 × 10^7^) of MCF-7 cells treated with PNA-antimiR-21 or the PNA-control were subcutaneously injected into female nude mice (eight animals per treatment). As seen in Figure 3b and 3c, detectable tumor masses (0.011 ± 0.013 g, mean ± standard deviation, SD) were seen in only 5/8 (62.5%) of mice in the MCF/PNA-antimiR-21 group, while much larger tumors (0.036 ± 0.038 g, mean ± SD) were detected in all mice in the MCF/PNA-control group (*P *= 0.065, Mann-Whitney test). Both tumor weight and number showed that MCF/PNA-control cells formed larger tumors more rapidly (Figure 3a, c) than MCF/PNA-antimiR-21 cells in nude mice. PNA-antimiR-21 reduced *miR-21 *expression by 5.72 log_2_-scale in MCF-7 cells 48 h post-treatment compared with that in the control (*P *< 0.01). *miR-21 *expression in xenograft tumors of PNA-antimiR-21 group was 0.96 log_2_-scale higher than that of the control group (*P *< 0.05; Figure 3e). Notably, MCF/PNA-antimiR-21 tumor cells were decreased in mitotic and pathological mitotic stages compared with MCF/PNA-control cells, indicating a decreased in cell proliferative activity and apoptosis (Figure 3d).

### Identification of potential *miR-21 *targets

**Figure 4:**
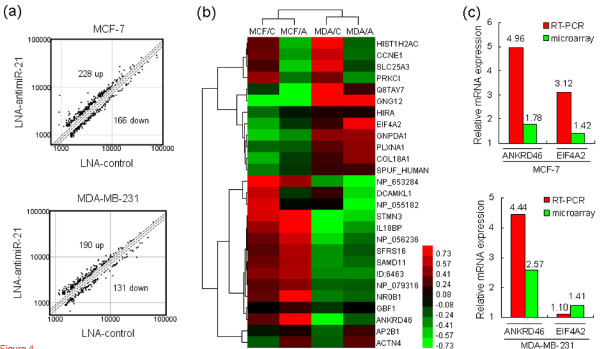
**mRNA profiling of miR-21-knockdown BC cell lines**. MCF-7 and MDA-MB-231 cells were transfected with 50 nM LNA-antimiR-21 or LNA-control. Total RNAs were isolated from MCF-7 cells 48 h post transfection and from MDA-MB-231 cells 36 h post transfection, respectively, and were hybridized to the human genome oligo array V1.0 (CapitalBio, China). **(a) **The scatter plot shows the LNA-antimiR-21 expression values normalized to LNA-control values, applying a cut-off of 1.3. Each dot represents one probe set. **(b) **Unsupervised hierarchical cluster analysis of intersection of mRNAs differentially expressed in MCF-7 and MDA-MB-231 cells upon LNA-antimiR-21 treatment. Rows, mRNAs; columns, biological samples. For each mRNA, red is expression higher than average expression across all samples, green is expression lower than average. **(c) **Validation of microarray results by qRT-PCR, normalizing on *GAPDH *RNA levels. MCF/A, MCF/LNA-antimiR-21; MCF/C, MCF/LNA-control; MDA/A, MDA/LNA-antimiR-21, MDA/C, MDA/LNA-control.

It is known that animal miRNAs regulate gene expression by inhibiting translation and/or by inducing degradation of target. In our study, most modulated genes in the mRNA differential expression profiles changed by less than two-fold. Since mRNA levels regulated by less than two-fold may still be miRNA targets, we defined differentially expressed genes as no less than 1.3-fold change [31]. Comparative analysis of LNA-antimiR-21 and LNA-control mRNA profiles showed differential regulation of 394 genes in MCF/LNA-antimiR-21, of which 228 (58%) were up-regulated and 166 (42%) were down-regulated. 321 genes were differentially expressed in MDA/LNA-antimiR-21 cells, of which 190 (59%) were up-regulated, and 131 (41%) down-regulated (Figure 4a). The intersection of MCF/LNA-antimiR-21 and MDA/LNA-antimiR-21 consisted of 27 genes (18 up- and 9 down-regulated; Figure 4b and Table 2).

**Table 2:** Regulated mRNAs in BC cells after *miR-21 *knockdown determined by oligo array

				Fold change		
					
Gene Name	NCBI accession number	Genbank accession number	Description	MCF-7	MDA-MB-231	Prediction program
***ANKRD46***	**NM_198401**	U79297, BC035087	**ankyrin repeat domain 46**	**1.78**	**2.57**^**#**^	**miRBase Targets V5**
*ACTN4*	NM_004924	BC005033	Alpha-actin 4 (F-actin cross linking protein)	1.75	1.55	
*NR0B1*	NM_000475	S74720	Nuclear receptor 0B1 (Nuclear receptor DAX-1)	1.60	1.32	
*HIRA*	NM_003325	BC039835, X89887	HIRA protein	1.48	1.32	
*COL18A1*	NM_030582, NM_130444, NM_130445	AF018081	Collagen alpha 1(XVIII) chain precursor	1.43	1.35	
*SAMD11*	NM_152486	BC024295, AK054643	sterile alpha motif domain containing 11	1.42	1.34	
***EIF4A2***	**NM_001967**	AL117412	**Eukaryotic initiation factor 4A-II**	**1.42**	**1.41**	**miRNAMap**
*SFRS16*	NM_007056	AF042800, AK094681	Splicing factor, arginine/serine-rich 16	1.40	1.34	
*NP_079316*	-	BC004930	zinc finger protein 614	1.37	1.36	
*IL18BP*	NM_173043, NM_005699, NM_173042	AF110801	Interleukin-18 binding protein precursor	1.36	1.34	
*GBF1*	NM_004193	AK025330, AF068755	Golgi-specific brefeldin A-resistance guanine nucleotide exchange factor 1	1.35	1.51	
*GNPDA1*	NM_005471	AJ002231	Glucosamine-6-phosphate isomerase	1.34	1.32	
*SPUF_HUMAN*	NM_013349	-	SPUF protein precursor	1.34	1.31	
*PLXNA1*	NM_032242	AK127254	plexin A1; plexin 1	1.32	1.57	
*STMN3*	NM_015894	AK094112	Stathmin 3 (SCG10-like protein)	1.32	1.47	
*NP_056236*	NM_015421	AK093383, AL080088	DKFZP564K2062 protein	1.31	1.33	
-	-	AK056473	-	1.31	1.30	
*AP2B1*	NM_001282	M34175	adaptor-related protein complex 2, beta 1 subunit	1.31	1.45	
*HIST1H2AC*	-	CR608156	Histone H2A.l (H2A/l)	-1.47	-1.67	
*PRKCI*	NM_002740	L33881, BC042405, BC022016	Protein kinase C, iota type	-1.43	-1.32	
*SLC25A3*	NM_002635, NM_213611, NM_213612, NM_005888	BX647062, AK057575	Phosphate carrier protein, mitochondrial precursor (PTP)	-1.39	-1.39	
*CCNE1*	NM_057182, NM_001238	M74093, BC035498	G1/S-specific cyclin E1	-1.37	-1.39	
*DCAMKL1*	NM_004734	AB002367	Serine/threonine-protein kinase DCAMKL1	-1.34	-1.46	
*GNG12*	NM_018841	AL832431	Guanine nucleotide-binding protein G(I)/G(S)/G(O) gamma-12 subunit	-1.32	-1.40	Miranda
*Q8TAY7*	NM_024869	BC025658	hypothetical protein FLJ14050	-1.32	-1.31	
*NP_653284*	NM_144683	BC015582	hypothetical protein MGC23280	-1.31	-1.35	
*NP_055182*	NM_014367	-	growth and transformation-dependent protein	-1.30	-1.34	

Fold-regulation of mRNAs affected by *miR-21 *knockdown compared with control-transfected cells (MCF-7 and MDA-MB-231) at indicated time points. Fold-changes for all mRNAs are derived from a single microarray experiment, with two relevant genes confirmed by qRT-PCR (Figure 4B). Shown are mRNAs changing by at least 1.3-fold in both cell lines. Genes in bold were selected for candidate gene analyses by qRT-PCR.; ^# ^marginal signal intensity; NCBI, National Center for Biotechnology Information; GB, GenBank.

To further screen potential direct targets, we compared the 27 candidate mRNAs with *miR-21 *targets predicted by TargetScan 5.1, miRBase Targets V.5, miRNAMap 2.0, PicTar and miRanda 3.0. Of the 27 mRNAs, 3 were recognized by the algorithms (Table 2). Because *miR-21 *targets are expected to up-regulated for the LNA-antimiR-21 samples, *ANKRD46 *and *EIF4A2*, the two up-regulated genes upon *miR-21 *knockdown in the two cell lines and predicted by target prediction programs, were selected for further investigation.

### *miR-21 *directly targets *ANKRD46 *in BC cells

**Figure 5:**
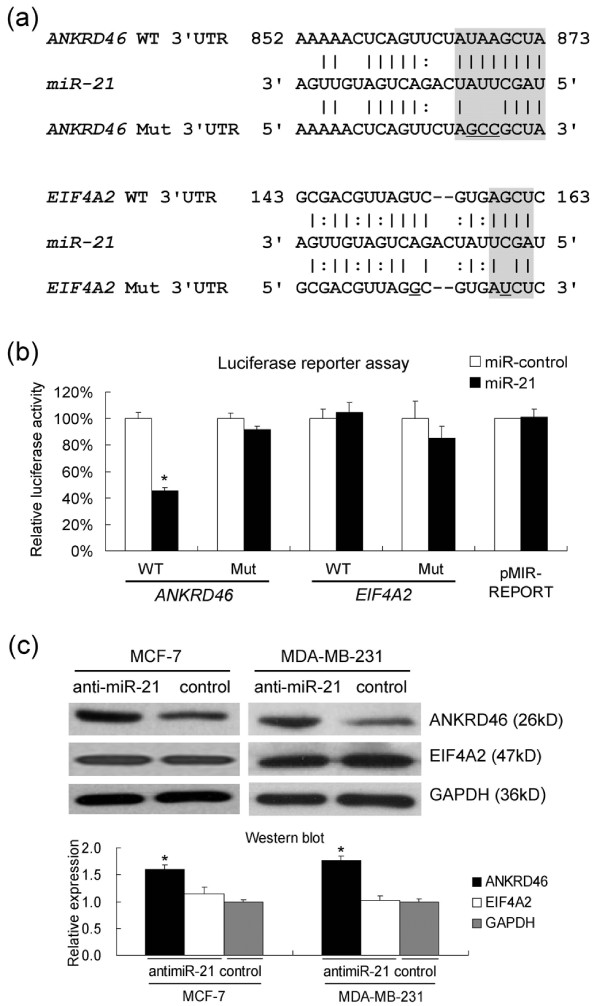
***ANKRD46 *is a direct target of *miR-21 *in BC cell lines**. **(a) **Predicted alignment of *miR-21 *with the target site derived from *ANKRD46 *and *EIF4A2 *3' UTR, determined with the software miRBase Targets V5 and miRNAMap, respectively. Note the seed matches at the 5' end of *miR-21 *(grey boxes) and the mutated nucleotides (underlined). **(b) **Luciferase assays show that *miR-21 *directly repress *ANKRD46 *mRNAs through 3' UTR interactions. Part of the 3' UTRs of wild-type *ANKRD46 *(478-bp length) and *EIF4A2 *(194-bp length), or the mutations were cloned into pMIR-REPORT vector (Applied Biosystems), downstream of luciferase. These vectors were then cotransfected with synthetic *miR-21 *(*pre-miR-21*) or miR-control in 293T cells, and luciferase activity was quantified. The graph shows the percentage of remaining luciferase activity calculated by normalizing the *miR-21 *expression values on the miR-control values. **(c) **Western blot to assay ANKRD46 and EIF4A2 after *miR-21 *knockdown, with GAPDH as equal loading control, followed by densitometric analysis. Cells were treated and harvested as described in Figure 4b. Data in **(b) **and **(c) **are mean + SD (*n *= 3). * *P *< 0.05. WT, wild-type; Mut, mutant.

The microarrays were validated by qRT-PCR assay. Consistent with the microarray results, qRT-PCR showed increased *ANKRD46 *and *EIF4A2 *mRNA levels in MCF-7 and MDA-MB-231 cell lines upon *miR-21 *inhibition, although the increase of *EIF4A2 *in MDA/LNA-antimiR-21 cells was relatively modest (Figure 4c). To determine whether *miR-21 *affects the expression of the potential endogenous target genes, we transfected MCF-7 and MDA-MB-231 cells with LNA-antimiR-21 or LNA-control. Western bolt showed that LNA-antimiR-21 led to up-regulation of endogenous ANKRD46 in both MCF-7 (*P *= 0.005) and MDA-MB-231 cells (*P *= 0.004), but no significant up-regulation of EIF4A2 protein (Figure 5c).

We further tested whether *miR-21 *could directly repress the identified mRNA targets through 3' UTR interactions (Figure 5a). Thus, the full-length 3' UTRs of the human genes *ANKRD46 *and *EIF4A2 *were cloned into the downstream of the luciferase gene (pMIR-REPORT), respectively. These vectors were then used to assess whether *miR-21 *could repress luciferase activity in 293T cells. *ANKRD46 *3' UTR showed a reduction to 54.8% of total luciferase reporter activity, in presence of *miR-21*, but *EIF4A2 *3' UTR did not display significant reduction of luciferase levels, compared with the miR-control (Figure 5b). These results suggest that *miR-21 *directly targets *ANKRD46 *in BC cells.

### *miR-21 *and EIF4A2 proteins are inversely expressed in resected patient tumors *in vivo*

**Figure 6:**
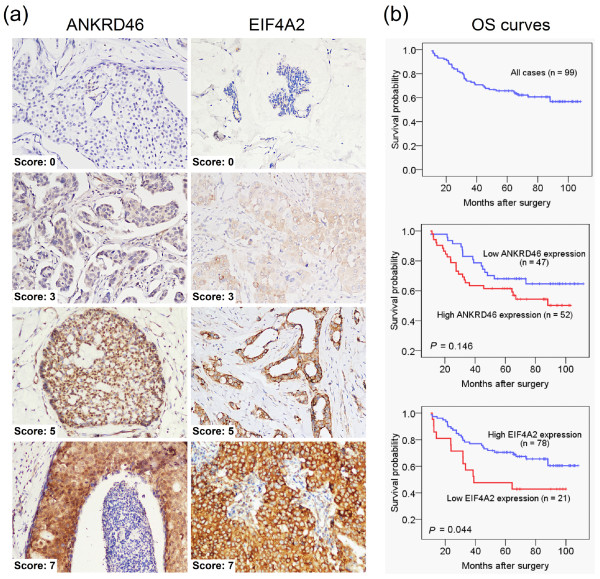
**EIF4A2 or ANKRD46 expression and survival in BC patients**. **(a) **Representative TMAs sections stained for ANKRD46 and EIF4A2 from different BC. Examples for staining score 0 (negative), 3, 5 and 7 of marker expression are shown. All images were taken at × 200. **(b) **Kaplan-Meier survival curve and log-rank test for 99 BC patients showing five-year overall survival. ANKRD46 expression had no significant relationship to patient survival (*P *= 0.146). High EIF4A2 expression was associated with significantly improved OS (*P *= 0.044) in women with BC. TMAs, tissue microarrays; OS, overall survival.

We examined ANKRD46 [NCBI: NP940683] and EIF4A2 [NCBI: NP001958] protein levels by IHC on TMAs constructed by the BC cases described in Materials and Methods. EIF4A2 was found in the cytoplasm, and ANKRD46 was seen in both the cytoplasm and nucleus (Figure 6a). ANKRD46 low expression was found in 47.5% (staining score < 4; media*n *= 4); EIF4A2 low expression was found in 21.2% (staining score < 7; median = 7) of the 99 BC cases. Next, we investigated the negative regulation of endogenous EIF4A2 and ANKRD46 protein by endogenous *miR-21 in vivo*. In 99 successfully tested cases out of 113, endogenous *miR-21 *and EIF4A2 protein levels were inversely expressed in resected patient tumors (r_s _= -0.283, *n *= 99, *P *= 0.005, Spearman's correlation analysis). However, no significant association between *miR-21 *and ANKRD46 (*P *= 0.181, Spearman's correlation analysis) was observed.

### Correlation of ANKRD46 and EIF4A2 expression with BC clinicopathological features and prognosis

Using Pearson's Chi-Square test, we found that, in BC tissues, IF4A2 protein correlated with CerbB2 status (*P *= 0.019), and ANKRD46 protein correlated with ER (*P *= 0.021) and PR (*P *= 0.001, Table 3). No significant correlation was observed between EIF4A2, ANKRD46, or other parameters. The five-year overall survival rate of the 99 BC patients was 59.60% (Figure 6b). The five-year survival rate in patients with high EIF4A2 protein level was 64.10% (*n *= 78), significantly higher than those with a low EIF4A2 protein level (42.86%, *n *= 21; *P *= 0.044, log-rank test; Figure 6b). The five-year survival rate in patients with high ANKRD46 protein was 53.85% (*n *= 52), which was not statistically different from those with low ANKRD46 protein (65.96%, *n *= 47; *P *= 0.146, log-rank test; Figure 6b).

**Table 3:** Correlation between target gene protein levels and clinicopathological parameters of 99 BC cases

Variable	EIF4A2			ANKRD46		
		
	Low (*n *= 21)	High (*n *= 78)	P*	Low (*n *= 47)	High (*n *= 52)	P*
Age (years)						
< 48	8	39	0.332	23	24	0.782
≥ 48	13	39		24	28	
Pathologic grade						
I	2	20	0.115	14	10	0.221
II, III	19	58		33	42	
Clinical stage**						
I, II	15	61	0.514	41	37	0.051
III	6	17		6	15	
Lymph node status						
Metastasis	14	43	0.342	23	34	0.098
No Metastasis	7	35		24	18	
ER status						
Negative	12	36	0.371	18	32	0.021
Positive	9	42		29	20	
PR status						
Negative	11	28	0.170	11	29	0.001
Positive	10	50		36	23	
CerbB2 status						
0,1+	17	41	0.019	29	18	0.832
2+,3+	4	37		31	21	

*Two-sided Pearson Chi-Square Test; ** there were no stage IV patients because all the cases selected were surgical patients without neoadjuvant therapy to avoid the effect of the preoperative radiotherapy and/or chemotherapy on miRNAs; EIF4A2, eukaryotic translation initiation factor 4A2 protein; ANKRD46, ankyrin repeat domain 46 protein; ER, estrogen receptor; PR, progesterone receptor; CerbB2, v-erb-b2 erythroblastic leukaemia viral oncogene homolog 2 receptors.

## Discussion

*miR-21 *is a key molecule in a wide range of cancers, and identifying its functional role in BC has direct clinical implications. We show here that knockdown of *miR-21 *suppresses cell growth and proliferation of MCF-7 cells *in vitro*, and suppresses MCF-7 xenograft growth. This result is consistent with the findings of Si *et al*. [9]. Interestingly, our study suggests that LNA-antimiR-21 also suppresses the growth and proliferation of MDA-MB-231 *in vitro*, in contrast to a recent report that found no effect of LNA-antimiR-21 on the growth of MDA-MB-231 *in vitro *or *in vivo*, although anti-miR-21-treated tumors were slightly smaller than control tumors [10]. One possibility could be differences in transfection efficiency, or miRNA ASO potency. Our results suggest that, as an oncomir, *miR-21 *also affects cell migration.

MCF-7 cells are hormone-sensitive and difficult to culture *in vivo*. Therefore, we used 17-estradiol to facilitate MCF-7 cells growth in nude mice, which is a common technique. Recently, estradiol was shown to down-regulate *miR-21 *expression in MCF-7 cells [32], although another study found estradiol-mediated up-regulation of *miR-21 *in MCF-7 cells [33]. In our study, the *miR-21 *knockdown effect was reduced from 5.72 log_2_-scale reduction before cell injection to 0.96 log_2_-scale reduction after mice sacrifice. Based on our results, we propose that estradiol reduced differences in *miR-21 *level between MCF/PNA-antimiR-21 and MCF/PNA-control cells, which would explain, in part, why differences in tumor weight between the two groups were not significance (*P *= 0.065). Nonetheless, treatment with anti-miR-21 reduced MCF-7 xenograft growth by approximately 68% for up to nine days. *In vivo *results suggested that the PNA-based *miR-21 *inhibitor had a subtle yet reproducible inhibitory effect on tumor growth. MCF-7 xenograft tumor sections demonstrated that *miR-21 *inhibition induced apoptosis of MCF-7 cells, confirming a previous study [9]. We also showed that miRNA inhibition can be achieved without transfection or electroporation of human BC cell lines, highlighting the potential of PNA for future therapeutic applications.

ANKRD46, also known as ankyrin repeat small protein (ANK-S), is a 228-amino acid single-pass membrane protein, of unclear function. For the first time, we identify *miR-21 *as an important regulator of *ANKRD46 *mRNA and protein levels in BC cells. Our data showed that *miR-21 *directly interacted with the *ANKRD46 *3' UTR and inhibited *ANKRD46 *expression, though there was no significant association between *miR-21 *and ANKRD46 in resected patient tumors. This discrepancy may be due to three reasons. First, the artificial luciferase reporter assays do not fully recapitulate miRNA regulation *in vivo *[34]; second, the expression of ANKRD46 protein in patient tumors reflected specific time-point feature, which maybe different to the *in vitro *subsequent increase of ANKRD46 protein at the time point of observation (the indicated hours after transfection); third, immunohistochemistry (IHC) is conventional a semi-quantitative method with relatively limited sensitivity. IHC may not be sensitive enough to observe the down-regulation of *ANKRD46 *by *miR*-21. Functional study of *ANKRD46 *is required in the future to determine weather *ANKRD46 *is a functional target of *miR-21 *in BC progression as demonstrated in this study.

*EIF4A2*, an ATP-dependent RNA helicase, is expressed widely in human tissues [35]. In this study, we found that *miR-21 *and EIF4A2 protein were inversely expressed in resected BC patient tumors. But we did not find *miR-21 *binding sites in the *EIF4A2 *3' UTR and found no significant increase of EIF4A2 protein upon *miR-21 *knockdown in MCF-7 and MDA-MB-231 cells, although *EIF4A2 *mRNA increased after anti-miR-21 transfection. Taken together, the data reported here suggest that there maybe unknown indirect interactions between *miR-21 *and *EIF4A2 *in BC progression. In adult mice, the expression of the two EIF4A isoforms is dependent on cell growth status, with EIF4A1 expressed in all tissues, while EIF4A2 is expressed only in tissues with a low rate of cell proliferation [36], indicating an anti-proliferative effect for EIF4A2. We for the first time revealed that low EIF4A2 expression correlated with low ERBB2 expression and poor survival of BC patients, suggesting its possible functional role in BC and urging further investigation.

## Conclusions

We demonstrate that MCF-7 and MDA-MB-231 cells transfected with anti-miR-21 show growth inhibition *in vitro *and *in vivo*, as well as cell migration *in vitro*. In addition, *ANKRD46 *is newly identified as a direct target of *miR-21 *in BC. These results suggest that *miR-21 *inhibitory strategies using PNA-antimiR-21 may have potential for therapeutic applications in BC treatment.

## Abbreviations

3' UTR: 3' untranslated region; AJCC: American Joint Committee on Cancer; ANK-S: ankyrin repeat small protein; ASOs: antisense oligonucleotides; BC: breast cancer; CerbB2: v-erb-b2 erythroblastic leukaemia viral oncogene homolog 2 receptors; CISH: chromogenic *in situ *hybridization; DAPI: 4',6-diamidino-2-phenylindole; EIF4A2: eukaryotic initiation factor 4A: isoform 2; ER: estrogen receptor; F: forward primer; FA: fibroadenoma; FISH: fluorescein *in situ *hybridization; FITC: fluorescein isothiocyanate; GEO: Gene Expression Omnibus; IHC: immunohistochemistry; L: length; LNA: Locked nucleic acid; miRNAs: microRNAs; MTT: 3-(4,5-dimethylthiazol-2-yl)-2,5-diphenyltetrazolium bromide; Mut: mutation; NATs: normal adjacent tissues; NBT/BCIP: nitro blue tetrazolium/5-bromo-4-chloro-3-indolyl phosphate; NCBI: National Center for Biotechnology Information; OS: overall survival; PNAs: peptide nucleic acids; PR: progesterone receptor; qRT-PCR: quantitative reverse transcription-polymerase chain reaction; R: reverse primer; RQ: relative quantification; RT: reverse transcription primer; SABC-AP: alkaline phosphatase-conjugated strept-avidin-biotin complex; SD: standard deviation: SYSUCC: Sun Yat-sen University Cancer Center; TMAs: tissue microrrays; TNM: tumor-lymph node-metastasis; W: width; WT: wild-type.

## Competing interests

Miss Li Xu Yan and Mrs Yan Zhang are doctoral degree candidates; Miss Qi Nian Wu and Mrs Yang Yang Li are master's degree candidates at SYSUCC. Mr Ding Zhun Liao, Mr Jing Hui Hou and Mrs Jia Fu are technicians at SYSUCC. Dr Mu-Sheng Zeng, Jing Ping Yun, Qiu Liang Wu and Yi Xin Zeng are Professors at SYSUCC. Dr Shao is a Professor and Vice Director at Department of Pathology of SYSUCC. The authors declare that they have not received any reimbursements, fees, funding, or salary, nor hold any stocks or shares in an organization that may in any way gain or lose financially from the publication of this manuscript, either now or in the future. The authors do not hold or are not currently applying for any patents relating to the content of the manuscript. The authors declare that they do not have any other financial or non-financial competing interests.

## Authors' contributions

LXY, QNW and YZ carried out the substantial experiment work and drafted the manuscript. JYS designed and financially supported the study. QNW and DZL were responsible for patient samples and tissue array construction. JHH and JF supported the immunohistochemistry. YYL carried out the luciferase reporter assay. JPY, MSZ, QLW and YXZ helped carry out the research design and critically reviewed the final version of the manuscript for submission. All authors read and approved the final manuscript.

## Supplementary Material

Additional file 1 **Word document containing the protocol of LNA-based CISH and FISH for miRNA in the present study**. Click here for file

Additional file 2 **Excel file containing a table listing RT and PCR primers for *miR-21 *and putative target genes**. Click here for file

Additional file 3 **PDF file comprising a figure showing the expression of *miR-21 *in human BC tissues and FA tissues by FISH**. Positive *in situ *hybridization signals are green (FITC). *miR-21 *expression levels in BC tissues are much higher than that in corresponding NATs. Click here for file

Additional file 4 **PDF file comprising a figure showing relative *miR-21 *induction by LNA-antimiR in BC cells**. Relative *miR-21 *induction in LNA-antimiR-21 transfected MCF-7 **(a) **and MDA-MB-231 cells **(b)**. Cells were transfected either with LNA-antimiR-21 or with LNA-control using Lipofectamine 2000 (Invitrogen) at indicated doses, and harvested at 12 h, 24 h, 36 h, 48 h, 60 h and 72 h, respectively. Total RNAs were isolated to quantify *miR-21 *expression by relative qRT-PCR, normalizing on *U6 RNA *levels. The graph shows a log_2_-scale RQ calculated by normalizing the *miR-21 *expression values in the LNA-antimiR-21 transfected cells on that in the LNA-control independently for each time point and dose. 50 nM LNA reagents at 48 h for MCF-7 and 50 nM at 36 h for MDA-MB-231 cells showed the best inhibition effects. Data indicate the mean (+SD) of three independent transfection experiments. Click here for file
