# Supplementary material for: Knockdown of miR-21 in human breast cancer cell lines inhibits proliferation, in vitro migration and in vivo tumor growth
Source: Breast Cancer Res. 2011 Jan 10;13(1):R2. doi: 10.1186/bcr2803 (PMC3109565; doi:10.1186/bcr2803)
Supplement: Additional file 1 — Word document containing the protocol of LNA-based CISH and FISH for miRNA in the present study. [file bcr2803-S1.DOC]

**Additional file 1**

**LNA-based CISH for miRNA.** LNA-modified oligonucleotide probe technology with a 3' fluorescein isothiocyanate (FITC) moiety (Exiqon A/S, Skelstedet, Vedbaek, Denmark) was combined with alkaline phosphatase-conjugated strept-avidin-biotin complex (SABC-AP) technology. Briefly, tissues were frozen in Tissue-Tek O.C.T. reagent (Sakura Finetechical Co.Ltd., Tokyo, Japan). Cryo-sections (8 µM) were fixed, acetylated and pre-hybridized in hybridization solution (50% formamide, 5×SSC, 0.5 mg/ml yeast tRNA, 1×Denhardt’s solution, DEPC-treated water) at 53°C. FITC-labeled miRNA LNA detection probe or scramble-miR as control (2.5 pmoles) was hybridized for 3 h at 53°C. After post-hybridization washing, slides were incubated with biotin-labeled anti-FITC (Sigma-Aldrich Corp, St. Louis, MO, USA) for 1 hour at 37°C. *In situ* hybridization signals were detected using the Enhanced Sensitive ISH Detection Kit (AP) (Boster, Wuhan, Hubei, China) followed by nitro blue tetrazolium/5-bromo-4-chloro-3-indolyl phosphate (NBT/BCIP) color reaction according to the manufacturer’s instructions. Methyl green was used to counterstain the nucleus. Slides were analyzed with an Olympus BX51 microscope equipped with a DP72 camera and DP2-BSW software (Olympus, Tokyo, Japan).

**LNA-based FISH for miRNA.** Cell suspensions (0.5 x 10^6^, 200 µl) were loaded in each cuvette, centrifuged at 2000 rpm for 3 min, and proceed with immediate fixation. Cryo-section preparation and hybridization procedure was as described above. After post-hybridization washes, slides were incubated with anti-FITC/HRP antibody (DAKO, Glostrup, Denmark) for 1 hour at 37°C. *In situ* hybridization signals were detected using the tyramide signal amplification system (PerkinElmer, Waltham, MA, USA) according to the manufacturer’s instructions. Slides were mounted in Prolong Gold containing 4',6-diamidino-2-phenylindole (DAPI) (Invitrogen) and analyzed with an Olympus BX51 microscope equipped with a Jenoptik camera and VideoTesT-FISH 2.0 software (Olympus).
