# Supplementary figures and images for: Knockdown of miR-21 in human breast cancer cell lines inhibits proliferation, in vitro migration and in vivo tumor growth
Source: Breast Cancer Res. 2011 Jan 10;13(1):R2. doi: 10.1186/bcr2803 (PMC3109565; doi:10.1186/bcr2803)

X 100

X 400

X 1000

BC

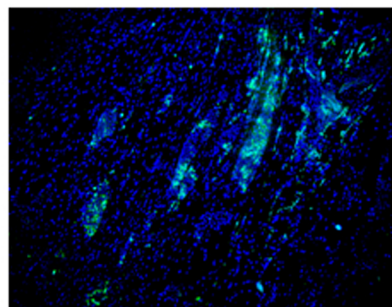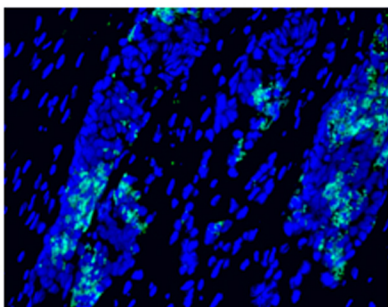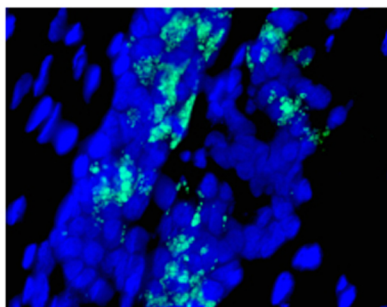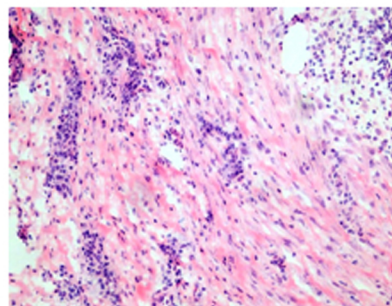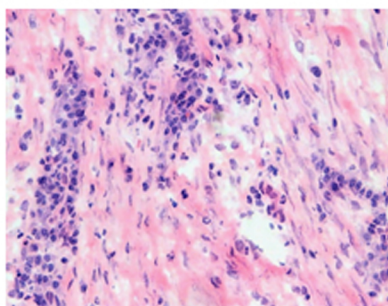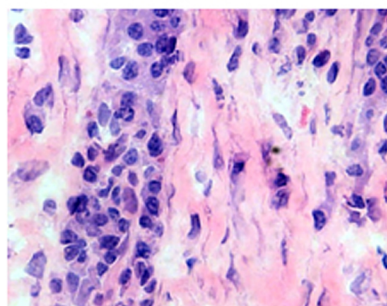

NATs

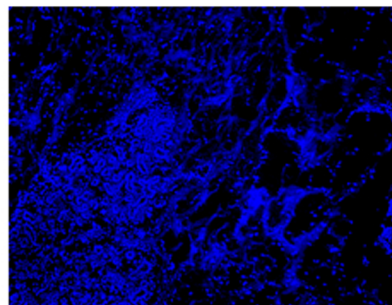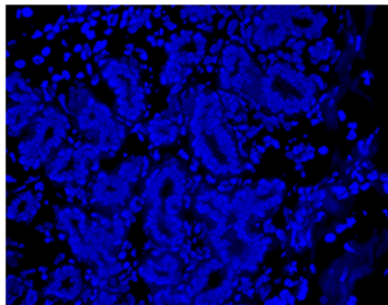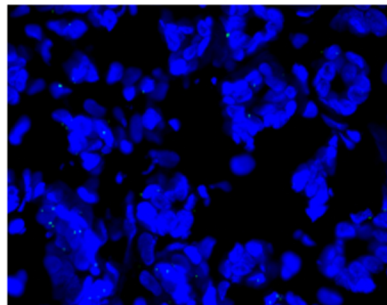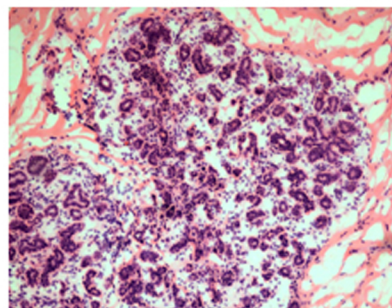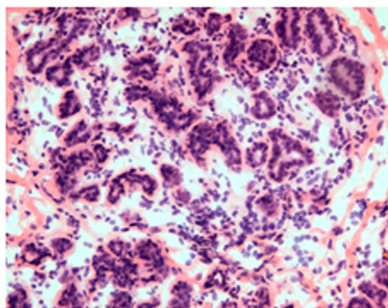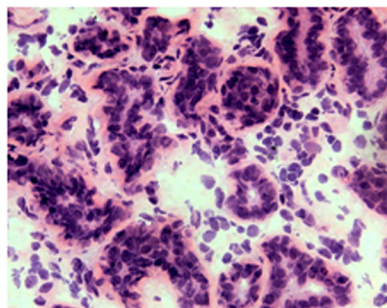

Supplement: Additional file 3 — PDF file comprising a figure showing the expression of miR-21 in human BC tissues and FA tissues by FISH. Positive in situ hybridization signals are green (FITC). miR-21 expression levels in BC tissues are much higher than that in corresponding NATs. [file bcr2803-S3.PDF]

(a)

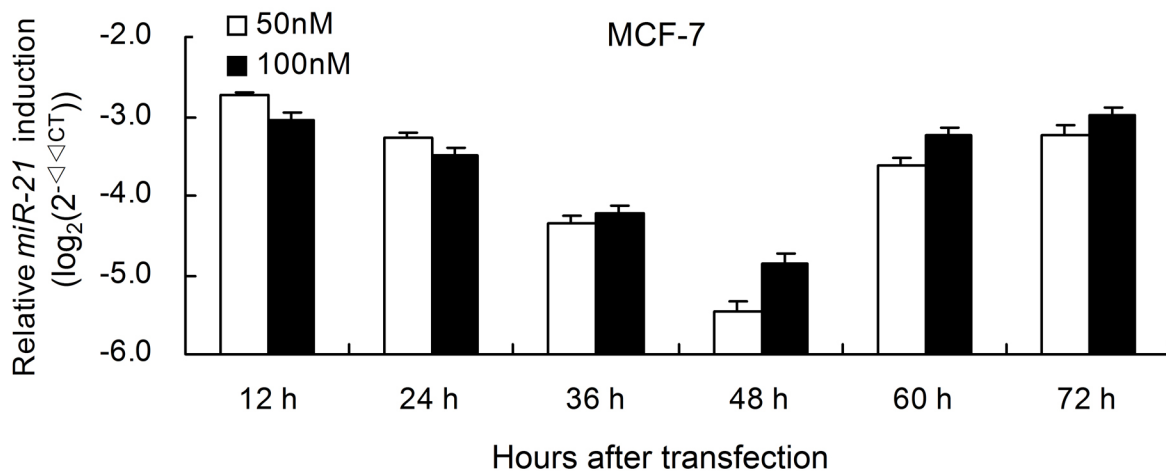

(b)

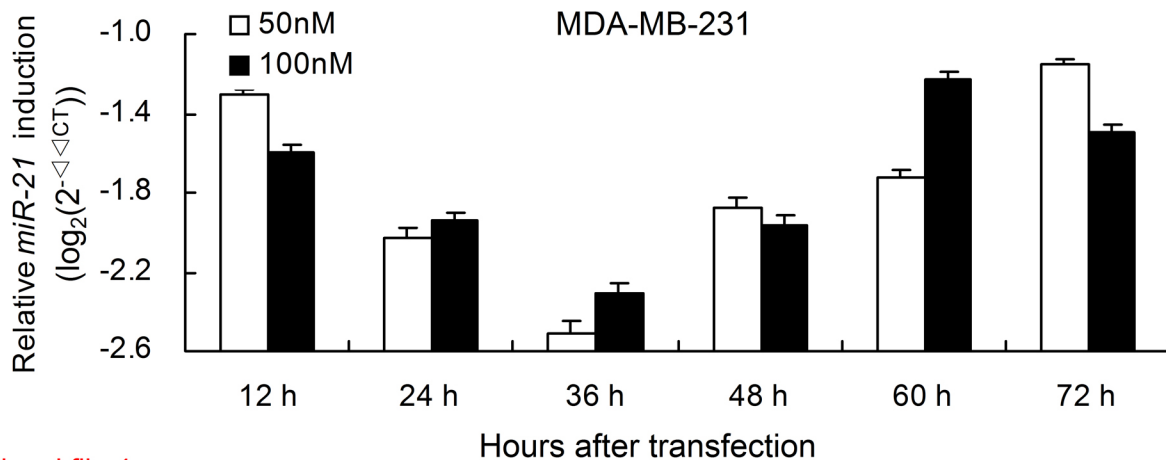

Supplement: Additional file 4 — PDF file comprising a figure showing relative miR-21 induction by LNA-antimiR in BC cells. Relative miR-21 induction in LNA-antimiR-21 transfected MCF-7 (a) and MDA-MB-231 cells (b). Cells were transfected either with LNA-antimiR-21 or with LNA-control using Lipofectamine 2000 (Invitrogen) at indicated doses, and harvested at 12 h, 24 h, 36 h, 48 h, 60 h and 72 h, respectively. Total RNAs were isolated to quantify miR-21 expression by relative qRT-PCR, normalizing on U6 RNA levels. The graph shows a log2-scale RQ calculated by normalizing the miR-21 expression values in the LNA-antimiR-21 transfected cells on that in the LNA-control independently for each time point and dose. 50 nM LNA reagents at 48 h for MCF-7 and 50 nM at 36 h for MDA-MB-231 cells showed the best inhibition effects. Data indicate the mean (+SD) of three independent transfection experiments. [file bcr2803-S4.PDF]
